# Demographic and clinical profiles of residents in long-term care facilities in South Africa: A cross-sectional survey

**DOI:** 10.4102/phcfm.v14i1.3131

**Published:** 2022-03-18

**Authors:** Letasha Kalideen, Jacqueline M. van Wyk, Pragashnie Govender

**Affiliations:** 1 School of Clinical Medicine, College of Health Sciences, University of KwaZulu-Natal, Durban, South Africa; 2 Department of Occupational Therapy, College of Health Sciences, University of KwaZulu-Natal, Durban, South Africa

**Keywords:** older people, demographic profile, socioeconomic profile, clinical profile, long-term care facilities, geriatric, ageing, South Africa

## Abstract

**Background:**

The demand for long-term care facilities (LTCFs) amongst older people in South Africa (SA) is growing and there is insufficient information on the profile and healthcare needs of this population.

**Aim:**

This study was conducted to describe the demographic and clinical characteristics of residents in LTFCs in SA.

**Setting:**

Three LTCFs in eThekwini district.

**Methods:**

A cross-sectional design was used to collect data from a purposive sample of 102 (*N* = 204) residents. A structured questionnaire was used to collect demographic and clinical data. The data were entered into Microsoft Excel and analysed descriptively and inferentially using R version 3.5.1 software.

**Results:**

The majority of the residents (59.8%) were between 65 and 80 years (78.9 ± 8.1 years) and 74.5% were women. The residents were white people (91.1%), SA born (82.4%) and widowed (54.9%). English was the primary language (91.1%), with the majority being christian (52.0%). Some residents had a university education, were previously employed and are financially independent. Ninety-three percent had clinical conditions, each suffering from at least three clinical conditions. Hypertension (63.7%), high cholesterol (53.9%), arthritis (38.2%), depression (37.3%) were the most prevalent clinical conditions recorded amongst the residents. Most residents were assessed to be intermediately frail, at risk of malnutrition and had mild depression as based on the respective mean frailty-, nutrition-, and geriatric depression scores.

**Conc lusion:**

Residents in LTCFs in the eThekwini district are more likely to be white people; women, christian, widowed, intermediately frail and at risk of malnutrition.

## Introduction

A long-term care facility (LTCF) provides accommodation, rehabilitative, restorative and other diverse professional services to residents.^1^ A 2019 report noted that 8.1% of the estimated 56.5 million people in South Africa (SA) represent the elderly population (≥ 60 years).^2^ There is, however, a dearth of evidence of the proportion of individuals (≥ 65 years) resident in LTCFs. Nonetheless, the proportion of the elderly population resident in LTCF’s in SA appears to be increasing in a similar trend as evidenced in high-income countries.^3^ To illustrate, about 3.9% of individuals aged 65 years and older are estimated to be residents in LTCFs in the United States.^4,5^ Long-term care facilities may include nursing homes, rehabilitation facilities, inpatient behavioural health facilities and long-term chronic care hospitals.^1^

Residents of LTCFs may have varied demographic and clinical profiles that may influence their healthcare needs and general well-being. For instance, the National Centre for Health Statistics in the United States (US) reported that in 2015–2016, the majority of long-term care service users were aged 65 years or older, with nearly 84% of nursing home residents being in that older age bracket with about 16% of residents younger than 65 years.^6^ The literature further indicates that residents in LTCFs are more likely to have compulsions, obsessions, delusions and auditory hallucinations.^7,8^ Additionally, residents in LTCFs may present with several other disease-related symptoms, including sleep disturbance, incontinence, depression, cognitive impairment, impulsiveness, anxiety, apathy, dysarthria, dysphagia, dystonia, weight loss, mutism, immobility and psychiatric conditions.^8,9,10,11^ Comorbid conditions such as hypertension, diabetes mellitus, arthritis and renal failure were also reported amongst LTCF residents.^8^

The diverse SA population represents many cultures; however, there are trends towards greater urbanisation and neglect of previous cultural norms and practices, such as home-based care for the elderly in one’s family. The SA apartheid policies also saw many indigenous people being forcefully removed from prime areas and further divided along racial categories which made access (after the development of prime land) in urban areas mostly unaffordable to persons from lower socio-economic groups (from whom the land had been confiscated in the first place).

The clinical and demographic profiles of residents from LTCFs in SA have not been well reported. A 2016 study conducted in Bloemfontein (SA) revealed that 72.1% of the 104 residents in the nursing home were women with an average age of 77 years.^12^ That study identified hypertension, joint disease, heart disease, cancer, psychological disorders and pain as the primary clinical conditions amongst elderly residents.^12^ A recent scoping review that explored the evidence on standards and quality of care for the elderly in LTCFs on the African continent found no published literature on this topic.^13^ However, the previously published literature relating to residents from LTCFs in SA reported on pathological long-bone fractures in residents with cerebral palsy^11^, social capital and mental well-being^12^, and cost-effective utilisation of nursing personnel in old age homes.^13^

To this end, this study provides a benchmark to report current evidence on the residential profile of people from LTCFs in 2021 in SA’s largest rural province, and the data can inform planning of healthcare services for older persons in similar settings. Therefore, this study described the demographic and clinical profiles of elderly residents from three LTCFs in the eThekwini district of KwaZulu-Natal (KZN) province.

## Methods

### Study design and setting

This study was a cross-sectional survey conducted amongst older residents of LTCFs in the eThekwini district, a Metropolitan health district and one of 54 municipalities in the KZN province.^14^ eThekwini has a developing population of about 3 548 512.^15^ The economically active group represents 64% of the total population, with children (< 15 years) and the elderly (> 60 years) accounting for 28% and 8%, respectively.^15^

The epidemiological disease profile of residents in eThekwini shows that the leading causes of death in the district include death due to injury or trauma, non-communicable disease (NCDs), human immunodeficiency virus (HIV) and tuberculosis (TB) and communicable diseases together with perinatal, maternal and nutritional conditions.^15^

The district service delivery platform in eThekwini is managed by municipal and provincial authorities.^15^ It consists of hospital level services provided by 17 hospitals, including one central hospital, one tertiary hospital, five regional hospitals, two district hospitals, one state-aided hospital, one eye specialist hospital, two specialised TB hospitals, one psychiatric hospital, two chronic hospitals and one hospital complex (inclusive of TB, dental, psychiatric and district services).^15^ There are eight community health centres (CHCs) and 103 primary health care (PHC) clinics. The PHC services are governed by different authorities (provincial and local government).^15^ Formally, there are 21 old age homes in eThekwini municipality.^16^

### Study population and sampling

The study population involved older persons who reside in three LTCFs (*N* = 204) in the eThekwini municipality of KZN province in SA. Three LTCFs were selected for this study because of lack of external funding and coronavirus disease 2019 (COVID-19) protocols that made many of these facilities completely locked in from the outside world. Both men and women aged 65 years and older (*n* = 102) were included. Individuals with advanced dementia, psychosis, comatose patients, medically unstable patients and residents who did not consent to the study were excluded. Firstly, three LTCFs were randomly selected from 21 LTCFs by simple random sampling. Of the 204 residents in the selected LTCFs, 102 residents met the eligibility criteria and consented. They were thus all included in the survey.

### Data collection

Data were collected from November 2020 to January 2021 using a structured questionnaire, and administered during a face-to-face interview by the principal investigator and a trained research assistant. The questionnaire was developed, piloted and amended (some questions were rephrased for clarity) after feedback. The questionnaire was developed using Microsoft Excel and administered via Google forms. The questionnaire included the following sections: (1) demographic data, (2) education and occupation, (3) personal and family history, (4) health status, (5) continence, (6) falls, (7) medical history, (8) health needs, (9) end-of-life planning, (10) screening (vision using International Functional Vision Screening Questionnaire,^17^ nutrition using Mini Nutritional Assessment: Nestle,^18^ and hearing using hearing handicap inventory – screening [HHIE-S] tool^19^), functional assessment (activities of daily living [ADL]), Instrumental Activities of Daily Living (IADL) using Lawton Scales,^20^ (11) frailty using Frail Scale,^21^ (12) depression using Geriatric Depression Scale,^22^ and (13) memory or cognition (mini mental state exam, MMSE).^23^

### Data analysis

The data were entered in Microsoft Excel and cleaned before analysis. The analysis was conducted using R version 3.5.1 and Microsoft Excel with the assistance of a statistician. Firstly, we performed a descriptive analysis of the sociodemographic and clinical profile of study participants, including demographic data, previous living area, education, employment, income sources, funding types, reasons for moving to the LTCF, health status, continence, falls, medical history, end of life planning and others. The resident’s vision was measured with 15 items (score ≥ 9 suggests a vision problem). Nutrition score was classified as follows: malnourished (0–7), at risk of malnutrition (8–11) and normal nutritional status (12–14). Hearing score was interpreted as no hearing impairment (0–8), mild-to-moderate hearing impairment (10–24) and significant hearing handicap (26–40). Activities of daily living score was classified as independent (ADL score = 6) and dependent (ADL score < 6). Instrumental activities of daily living score was interpreted as dependent (IADL score less than 8 for women and less than 5 for men) and independent (IADL score of 8 for women, and 5 for men). Frailty score was interpreted as not frail (score = 0), intermediate (score 1–2) and frail (score > 3). A geriatric depression score of 0–5 suggested mild depression, moderate depression (score > 5) and severe depression (score > 10). Cognitive impairment was categorised as follows: no cognitive impairment (MMSE score 24–30), mild cognitive impairment (MMSE score 18–23) and severe cognitive impairment (MMSE score 0–17). Subsequently, Rank sum test, Kruskal–Wallis test, Chi-square test or Fisher’s exact test were conducted to evaluate score differences (vision, nutrition, hearing, ADL, IADL, frailty, geriatric depression and MMSE), and comparisons were made between female and male participants. Spearman’s rank correlation was also performed to explore associations between the scores obtained. Results were statistically significant when the *p*-value was less than 0.05 (*p* < 0.05).

## Results

### Sociodemographic characteristics

#### Demographic characteristics

According to the demographic profile of the study participants, overall, a total of 102 elderly residents participated in the study (Table 1). The majority (82.4%) of the residents were born in SA (3.3% in KZN province) and 17.6% were born overseas. The proportion of female to male participants was at 3:1 (74.5% vs. 25.5%). The mean age of the residents was 78.9 years (standard deviation [s.d.] = 8.1 years), and most participants were aged between 65 and 80 years (59.8%). Most of the residents from the LTCFs were white (91.1%), with black residents (2.0%) being the least represented group. Christians were the majority 52.0%, whilst Hindu constituted the least (4.9%). The majority (54.9%) of them were widows or widowers, and the least 0.01% were separated.

**TABLE 1:** Demographic characteristics of the study participants.

Variable	Overall (*N* = 102)	
	*N*	%
**Place of birth**		
South Africa	84	82.4
Overseas	18	17.6
**Gender**		
Male	26	25.5
Female	76	74.5
**Age (years)**		
65–74	27	26.5
75–84	43	42.2
≥ 85	32	31.3
**Race**		
White people	93	91.1
Indian people	5	6.9
Black people	2	2.0
**Home language**		
English	93	91.1
Zulu	2	2.0
Afrikaans	2	2.0
Italian	2	2.0
Other (French, German, Hebrew)	3	2.9
**Religion**		
Christian	56	52.0
Jewish	43	42.1
Hindu	5	4.9
Other	1	1.0
**Marital status**		
Widow or widower	56	54.9
Married	28	27.5
Divorced	8	7.8
Never married	5	4.9
Single	3	3.9
Separated	1	1.0

#### Socioeconomic characteristics

Of the 102 participants, 30.4% had university education, whilst 1.0% had no formal education. Per their previous employment, 46.1% of the 102 participants worked in the formal sector as a professional, whilst 44.1% worked at the informal sector. Nearly 10.0% of the participants were never employed. Of the 102 participants, 76.5% were financially independent. According to their current source of income, 61.7% earn from pension schemes (39.2% private and 22.5% government pension); however, close to 7% (7/102) did not have any source of income. Most residents (73.5%) were admitted from their own homes, whilst the least (4.9%) were admitted from other types of accommodation. About 25% (26/102) of the residents relocated to the LTCF because of physical challenges, and few (4.9%) were relocated because of mental challenges. In terms of their duration of stay in the LTCF, almost half (48.0%) had stayed between one month and one year, and approximately 10% were within one-month duration. The majority (65.7%) of the 102 participants lived independently at the LTCF, whilst 7.8% were frail. About 77.5% of the study participants had family supporting them and 22.5% were not supported. Of the 102 study participants, most of them (52.9%) said their children resided in other countries and 14.7% did not have children. Fifty-two percent of the participants had multiple funding sources, including state pension and non-governmental organisations (NGOs), and the remainder 48% were funded privately (see Appendix 1, Table 1-A1 for the socioeconomic characteristics of the study participants).

### Clinical characteristics

**FIGURE 1:**
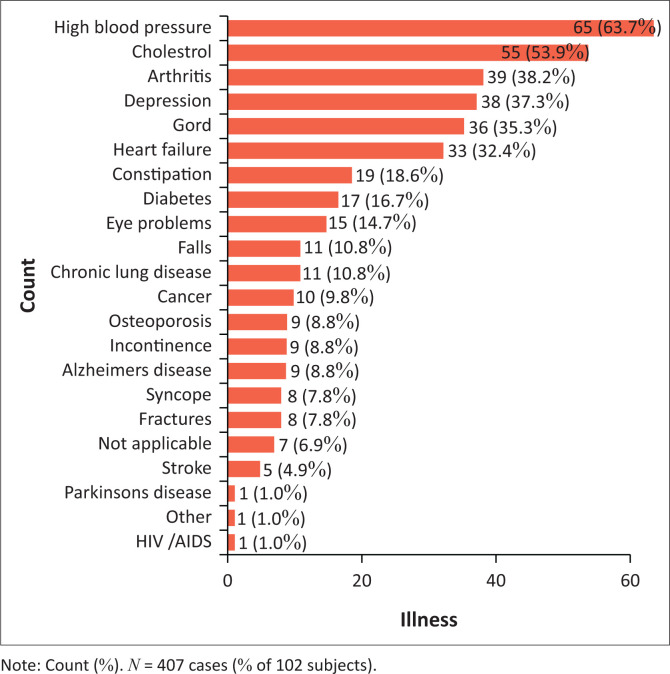
Distribution of clinical conditions of the study participants.

Of the 102 participants, about 93.1% had clinical conditions, each suffering from at least three clinical conditions. Out of the 407 cumulative conditions, the most prevalent clinical conditions recorded included hypertension (63.7%), high cholesterol (53.9%), arthritis (38.2%), depression (37.3%), gastro-oesophageal reflux disease (GORD) (35.3%) and heart failure (32.4%). Parkinson’s disease (1%) and HIV or AIDS (1.0%) were amongst the least prevalent clinical conditions recorded in the study (Figure 1).

About 86.3% of the 102 participants had a chronic illness and 95.0% of the residents were on chronic medications, with the remaining 5% not on any chronic medication. The chronic medication of approximately 94.0% (83/88) of residents was administered by a private doctor, and a clinic or hospital member of staff administered medication to the remaining 6.0%. The duration of being chronically ill was for more than three months in 99.0% (87/88) of the study participants, with the remaining 1.0% being diagnosed within 1 month prior to data collection. In terms of vaccination against flu, 63.7% (65/102) were not vaccinated. About 91.9% (34/37) were vaccinated against Influenza, and the remainder 8.1% against pneumococcal infection. Of the 36.3% (37/102) who had an influenza vaccine, 51.4% were vaccinated about two years ago (before 2019), whilst 13.5%, and 35.1% of the residents last had an influenza vaccine in 2019 and 2020, respectively.

**FIGURE 2:**
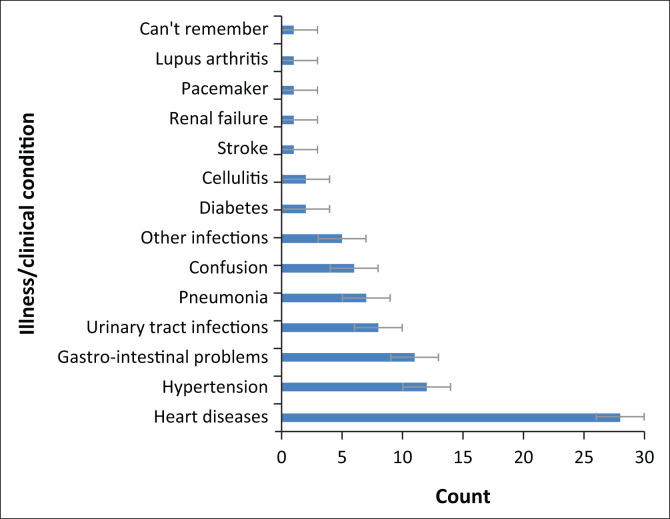
Cluster bar chart showing illnesses or clinical conditions accounting for hospitalisation (*n* = 86).

More than half (84.3%) of the 102 study participants were admitted to hospital at least once within the period of staying in the LTCF. Heart diseases accounted for most (about 32.6%) of the hospitalisation (Figure 2). The majority (61.2%) of them were last admitted to hospital more than a year ago. The duration of stay at the hospital range from one day to more than two weeks, with about 74.6% hospitalised for less than one week and the remainder 25.4% for more than one week. Most (87.3%) of the 102 participants accessed private medical services, and the remainder (12.7%) accessed government healthcare facilities.

Residents received varying levels of healthcare and health monitoring. Most residents (48.0%) received care from doctors, and 26.5% were cared for by both doctors and nurses. Doctors, nurses, and other caregivers cared for the remaining 25.5%. The majority (84.3%) of the 102 participants were visited by a health provider once every six months. Approximately 6.0% and 2.0%, respectively, were visited by a health provider annually and monthly, but 7.8% were not visited by health providers. Most residents knew about end-of-life planning. About 94.0%, 90.2% and 61.8% of the study participants, respectively, had knowledge about power of attorney, advance care directive, and both power of attorney and advance care directive.

Approximately 28.4% of the 102 residents had a history of incontinence, with 25.0% struggling with bladder incontinence and the remainder 3.4% with bladder and bowel. Difficulty in getting to the toilet is accounted for in 70.0% (21/30) of the participants by the incontinence, and the remainder 30.0% (9/30) were because of ingestion of sleeping tablets, arthritis and poor mobility. About 42.2% (43/102) had a history of falls within the last five years, of which 22.5% recorded one fall. Sixty-nine percent of the (30/43) residents who had a history of falls sustained various injuries, with nearly 53.4% (16/30) of the falls resulting in fractures.

The results of the nutritional assessment of the 102 residents were discussed, as shown in Table 2. For 68.6% of the 102 residents, the body mass index (BMI) was greater than 23. Nearly (75.0%) had no declined food intake, but 15.7% had severely reduced food intake over the last three months. Weight loss was not recorded for 80% of the residents, whilst 15.7% had lost more than 3 kg within the last three months. With regard to their mobility, psychosocial stress and neuropsychiatric assessment, respectively, 90.2% of them were able to go out, 27.5% were psychologically stressed, and 6.9% were severely demented or depressed.

**TABLE 2:** Nutritional assessment results.

Variable	Overall (*N* = 102)	
	*n*	%
**BMI**		
< 19	12	11.8
19 to < 21	4	3.9
21 to < 23	16	15.7
23+	70	68.6
**Declined food intake**		
None	76	74.5
Moderate	10	9.8
Severe	16	15.7
**Weight loss 3 months**		
None	80	78.4
1–3 kg	5	4.9
> 3 kg	16	15.7
Unknown	1	1.0
**Mobility**		
Able to get out of bed and chair	5	4.9
Bed or chair bound	5	4.9
Goes out	92	90.2
**Psychological distress**		
No	74	72.5
Yes	28	27.5
**Neuropsychiatric**		
Mild dementia	15	14.7
None	80	78.4
Severe dementia or depression	7	6.9

BMI, body mass index.

**FIGURE 3:**
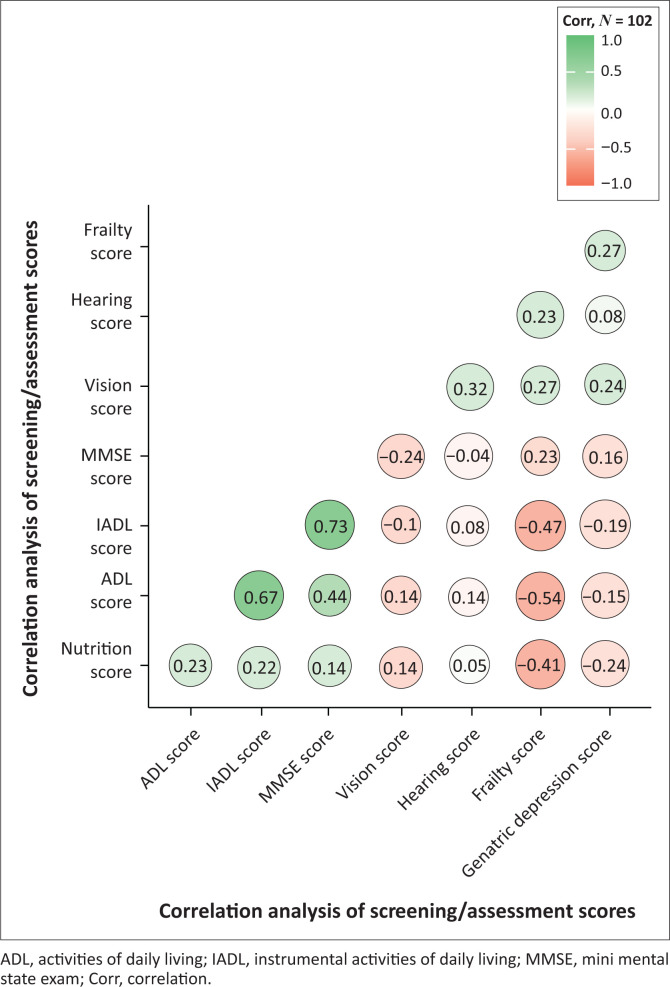
Spearman’s rank correlation analysis results.

The overall mean and standard deviation (s.d.) for the screening or assessment scores were vision (1.36 ± 2.89), nutrition (10.7 ± 2.97), hearing (6.53 ± 11.0), ADL (5.33 ± 1.37), IADL (6.38 ± 2.53), frailty (1.29 ± 1.34), geriatric depression (3.55 ± 3.65) and MMSE (27.3 ± 5.07). No significant differences were observed between men and women in terms of vision score (*p* = 0.783), nutrition score (*p* = 0.424), hearing score (*p* = 0.212), ADL score (*p* = 0.141), IADL (*p* = 0.429), frailty score (*p* = 0.675), geriatric depression (*p* = 0.843) and MMSE (*p* = 0.382) (Table 3). A strong positive correlation was observed between ADL and IADL scores (*r* = 0.666; *p* ≤ 0.001), IADL and MMSE scores (*r* = 0.728; *p* ≤ 0.001), and moderate correlation between ADL and MMSE scores (*r* = 0.444; *p* ≤ 0.001). However, a negative correlation was observed between the nutrition score and frailty score (*r* = –0.536; *p* ≤ 0.001; Figure 3).

**TABLE 3:** Comparison of vision, nutrition, hearing, activities of daily living, instrumental activities of daily living, frailty, geriatric depression and mini mental state exam score differences between women and men.

Variable	Female (*N* = 76)					Male (*N* = 26)					*p*	Overall (*N* = 102)				
	Mean ± s.d.	CV%	Median	Q1–Q3	Min–Max	Mean ± s.d.	CV%	Median	Q1–Q3	Min–Max		Mean ± s.d.	CV%	Median	Q1–Q3	Min–Max
**Vision score**	1.59 ± 3.19	200.5	0	0.00–1.00	0.0–12.0	0.692 ± 1.62	233.9	0	0.00–1.00	0.0–8.00	0.783	1.36 ± 2.89	212.3	0	0.00–1.00	0.0–12.0
**Nutrition score**	10.7 ± 2.94	27.5	12.0	9.75–13.0	2.00–14.0	10.9 ± 3.10	28.5	12.5	8.25–13.0	4.00–14.0	0.424	10.7 ± 2.97	27.6	12.0	9.00–13.0	2.00–14.0
**Hearing score**	5.87 ± 10.5	178.2	0	0–6.00	0–40.0	8.46 ± 12.6	148.3	2.00	0–9.50	0–36.0	0.212	6.53 ± 11.0	168.8	0	0–8.00	0–40.0
**ADL score**	5.24 ± 1.44	27.5	6.00	5.00–6.00	1.00–6.00	5.62 ± 1.10	19.6	6.00	6.00–6.00	2.00–6.00	0.141	5.33 ± 1.37	25.6	6.00	5.00–6.00	1.00–6.00
**IADL score**	6.21 ± 2.70	43.5	8.00	5.00–8.00	0–8.00	6.88 ± 1.88	27.4	8.00	6.25–8.00	2.00–8.00	0.429	6.38 ± 2.53	39.6	8.00	5.00–8.00	0–8.00
**Frailty score**	1.28 ± 1.36	106.7	1.00	0–2.00	0–5.00	1.35 ± 1.29	96.2	1.00	0–2.00	0–5.00	0.675	1.29 ± 1.34	103.5	1.00	0–2.00	0–5.00
**Geriatric depression score**	3.38 ± 3.30	97.7	2.00	1.00–5.25	0–13.0	4.04 ± 4.56	112.8	2.50	1.00–6.75	0–14.0	0.843	3.55 ± 3.65	102.8	2.00	1.00–6.00	0–14.0
**MMSE score**	27.0 ± 5.54	20.5	30.0	27.0–30.0	8.00–30.0	28.1 ± 3.29	11.7	30.0	26.5–30.0	20.0–30.0	0.382	27.3 ± 5.07	18.6	30.0	27.0–30.0	8.00–30.0

Note: *P*-values based on non-missing cases only. Test used: Rank sum test, Kruskal–Wallis test, Chi-square test and Fisher’s exact test.

ADL, activities of daily living; IADL, instrumental activities of daily living; MMSE, mini mental state exam; s.d., standard deviation; CV, co-efficient variant.

## Discussion

This study reports the demographic and clinical profile of older residents from three LTCFs in the eThekwini district of SA. The demographic profile highlights that most of the residents from LTCF in the eThekwini district are white, Christian, widowed women between 65 and 80 years of age. The residents were primarily persons with tertiary education, previously employed and financially independent. The findings reveal that most residents from three LTCFs in the eThekwini district relocated because of physical challenges, safety concerns, lack of help at home, financial difficulties, death of a spouse and mental challenges. Despite these reasons, most residents (65.7%) lived independently at the LTCF.

The demographic profile of residents in this study in terms of race, gender and age is similar to other previous studies conducted in SA.^12,24^ In SA, Gerber et al. study of older persons to determine health-related quality of life and functional abilities in nursing homes in Bloemfontein, similarly found that 72.1% of the 104 study participants were women with a mean age of 77 years.^12^ Also, this study’s findings on the sex and race of the residents were similar to those reported by Perold and Muller in their study, which investigated the composition of old age homes in SA.^24^ They found many of the residents in their study were women and mostly classified as white people (83, 74%).

Moreover, this study found that hypertension, high cholesterol, arthritis, depression, GORD and heart failure were the most prevalent clinical conditions reported amongst the residents. Similarly, this study findings on the clinical characteristics of the residents support the study results of the Gerber et al.^12^ Also, our findings on the clinical profile of the residents’ support those of Gerber et al. in their study in Bloemfontein.^12^ They reported that most of the study participants had a history of a minimum of two of the following diseases: hypertension, joint disease and heart disease.^12^ This is probably because of age similarities reported by both studies, and also, because of socioeconomic similarities.

This study showed that most residents were older than 77 years. Getting older can bring senior health challenges such as heart diseases, diabetes, falls, arthritis, cancer and others. An awareness of these common chronic conditions can assist relevant stakeholders, and LTCF can take steps to stave off disease or plan and implement essential interventions to reduce mortality or disability amongst older persons. To this end, placement assessment needs to be reviewed regularly to identify needs and appropriate interventions. This study found that the residents mostly belong to the white race. This may be because of cultural, socioeconomic and historical influences that affect (the use of LTCF or nursing homes), and if so, community education targetting other ethnic groups will be needed to change the stereotypes about the use, access, or benefit of LTCF. Greater proportions of the residents are widows, and hence, we recommend elder care clubs and community engagement and outreach programmes to address social isolation among the elderly, especially that almost 14% reported not receiving help at homes as the reason for relocating to the LTCF. Aside from this reason, about 53% of older people resident in LTCFs in this sample had their family living abroad. Therefore, it is essential to regularly evaluate and provide relevant psychological, physical and social support to residents, especially those who have their families living abroad. These psychological, physical and social support if provided, as well as creating mental health awareness, will help to facilitate their physical and psychological well-being at the LTCFs, especially approximately 7% of the residents suffered dementia or depression. However, it will be essential to first understand the unmet needs of the residents in order to plan and provide the needed psychological, physical and social support interventions. Based on their medical history, we recommend implementing preventive strategies, such as early and regular screening, for the detection and timely initiation of treatment or care to prevent complications because of hypertension, cholesterol, arthritis, depression, osteoporosis and GORD. These in conjunction with structured physical exercise programmes, physical rehabilitation and occupational therapy will be essential to reduce the sequelae or complications of the clinical conditions as reported in the study findings.

Moreover, the frailty and nutrition scores of the residents in this study suggest that they were intermediately frail and at risk of malnutrition. This potentially could place them at high risks of diseases or infections. Even more worrying is that the BMI of about 69% of the study participants was over 23. People who are obese, compared with those having a healthy weight, have a higher risk for many serious diseases and health conditions, including the following: mortality, hypertension, dyslipidaemia, type 2 diabetes, coronary heart disease, stroke, gallbladder disease and osteoarthritis.^25,26,27^ High BMI can additionally increase the risks of sleep apnoea and breathing problems; cancer; mental illness such as clinical depression, anxiety,^28,29^ other mental disorders; body pain and difficulty with physical functioning.^30^ Therefore, we recommend the education of residents, the LTCF managers, and the caregivers on diet, supplementation, and risk factors for frailty and vaccination of residents alongside a half-yearly review of their ADLs and IADLs.

This study’s participants were selected using a random method that allowed all eligible residents to be included in the survey. This study has provided a snapshot of the sociodemographic and clinical profile of older people residing on three LTCFs in eThekwini (SA). The results add to the body of knowledge on the demographic and clinical profile of elderly residents in SA’s LTCFs. Despite these strengths, there are numerous weaknesses for this study. This study involved only three private LTCFs within one district in the KZN province, and hence, it may not be generalised for the whole of SA. Also, the data for this study are cross-sectional and are subject to all limitations associated with the study design. Furthermore, this study’s eligibility criterion was limited to residents aged 65 years and above, which possibly excluded younger residents from the LTCFs. Nonetheless, this study has provided research evidence useful for baseline planning or strategising improvements of care, hypothesis generation and future research to improve the quality of care and life of older residents in LTCFs in the study area and elsewhere both nationally and abroad, where the population characteristics are similar to those in the eThekwini district. Beyond establishing the demographic and clinical characteristics of the residents, it may be worthwhile to understand the lived experiences of the residents in LTCFs as well as the care providers and managers’ experiences. This, together with the survey results, will provide a holistic understanding of the needs of the residents, care providers and the managers for reforms to improve the quality of life of the residents. To this end, we recommend that subsequent studies focus on the lived experiences of older persons resident in LTCFs and the experiences of caregivers and the LTCF managers using qualitative research methods.

## Conclusion

This research study provides insights into the socioeconomic and clinical characteristics of older residents of LTCF and their reasons for choosing to live there. Furthermore, the study highlights residents’ vulnerability to malnutrition, frailty and depression, and suggests urgent needs for clinical and non-clinical interventions to address these priority issues. A future qualitative exploration of lived experiences of residents at the LTCFs, those of the facility managers and caregivers would add great understanding of the quantitative results and their needs for necessary reforms to improve their quality of life whilst residing in the LTCF.

## APPENDIX 1

**TABLE 1-A1:** Socioeconomic characteristics of the study participants.

Variable	Overall (*N* = 102)	
	*n*	%
**Education**		
High school	62	60.8
University	31	30.4
Others	8	7.8
No formal education	1	1.0
**Employment**		
Formal sector	47	46.1
Informal sector (including self-employment)	45	44.1
No employment	10	9.8
**Financial independence**		
Yes	78	76.5
No	24	23.5
**Current income sources**		
Private pension	40	39.2
Government pension	23	22.5
Gifts or donations from families and/ or friends	12	11.8
Investments	10	9.8
Spouse’s income	3	2.9
Inheritance	2	2.0
Salary	2	2.0
Others (disability grant, assets and church elder)	3	2.9
No income	7	6.9
**Previous residential location**		
Urban or city	51	50.0
Township or suburb	50	49.0
Rural	1	1.0
**Previous accommodation status**		
Own home	75	73.5
Rented home	14	13.5
Family home	8	7.8
Others	5	4.9
**Reasons for moving to the LTCF**		
Physical challenges	26	25.5
Safety concerns	23	22.5
No help at home	14	13.7
Financial difficulty	11	10.8
Loss of spouse	6	5.9
Mental challenges	5	4.9
**Duration at the LTCF**		
≤ 1 month	10	9.8
> 1 month ≤ 1 year	49	48.0
> 1 year ≤ 5 years	16	15.7
> 5 years	27	26.5
**Family support**		
Yes	79	77.5
No	23	22.5
**Residential location of children**		
Outside South Africa	54	52.9
Within KZN province	4	3.9
Other provinces in South Africa	30	29.4
Not applicable	15	14.7
**Services provided in LTCF**		
Independent	67	65.7
Assisted	27	26.5
Frail	8	7.8
**Funding type**		
Multiple sources	53	52.0
Private	49	48.0

LTCF, long-term care facility; KZN, KwaZulu-Natal.

## References

[1] MedicineNet. Medical definition of long-term care facility [homepage on the Internet]. MedicineNet; 2021 [cited 2021 Jun 03]. Available from: https://www.medicinenet.com/long-term_care_facility/definition.htm

[2] Stats SA. Statistical release: Mid-year population estimates. Pretoria: Stats SA; 2019.

[3] Lloyd-Sherlock P. Long-term care for older people in South Africa: The enduring legacies of apartheid and HIV/AIDS. J Soc Policy. 2019;48(1):147–167. 10.1017/S0047279418000326

[4] OECD/European Commission. A good life in old age? Monitoring and improving quality in long-term care. OECD Health Policy Studies. 2013. 10.1787/9789264194564-en

[5] La Frenais FL, Bedder R, Vickerstaff V, Stone P, Sampson EL. Temporal trends in analgesic use in long-term care facilities: A systematic review of international prescribing. J Am Geriatr Soc. 2018;66(2):376–382. 10.1111/jgs.15238 29274247 PMC5838548

[6] Howley EK. Nursing home facts and statistics. U.S.NEWS 2020 Nov. https://health.usnews.com/health-news/best-nursing-homes/articles/nursing-home-facts-and-statistics

[7] Wheelock VL, Tempkin T, Marder K, et al. Predictors of nursing home placement in Huntington disease. Neurology. 2003;60(6):998–1001. 10.1212/01.WNL.0000052992.58107.67 12654967

[8] Zarowitz BJ, O’Shea T, Nance M. Clinical, demographic, and pharmacologic features of nursing home residents with huntington’s disease. J Am Med Dir Assoc. 2014;15(6):423–428. 10.1016/j.jamda.2014.01.010 24613270

[9] Nance M, Sanders G. Characteristics of individuals with Huntington disease in long-term care. Mov Disord. 1996;11(5):542–548. 10.1002/mds.870110509 8866495

[10] Setter S, Neumiller J, Dobbins E, et al. Treatment of chorea associated with Huntington’s disease: Focus on tetrabenazine. Consult Pharm. 2009;24(7):524–537. 10.4140/TCP.n.2009.524 19689181

[11] Seitz D, Purandare N, Conn D. Prevalence of psychiatric disorders among older adults in long-term care homes: A systematic review. Int Psychoger. 2010;22(7):1025. 10.1017/S1041610210000608 20522279

[12] Gerber AM, Botes R, Mostert A, Vorster A, Buskens E. A cohort study of elderly people in Bloemfontein, South Africa, to determine health-related quality of life and functional abilities. S Afr Med J. 2016;106(3):298–301. 10.7196/SAMJ.2016.v106i3.10171 26915946

[13] Kalideen L, Govender P, Van Wyk J. Standards and Quality of Care for the Elderly in Long Term Care Facilities: A Scoping Review I Mapping evidence on standards and quality of care for older persons in long-term care facilities: a scoping review protocol. Systematic Reviews. 2021 Dec;10(1):1–6. 34022957 10.1186/s13643-021-01709-2 PMC8141225

[14] South African Government. Provincial and local government directory: KwaZulu-Natal municipalities South Africa [homepage on the Internet]. South African Government; 2020 [cited 2020 Nov 02]. Available from: https://www.gov.za/about-government/contact-directory/kzn-municipalities?gclid=CjwKCAiA-f78BRBbEiwATKRRBKmqhveQTQx2DLiTUIwj54PY3L6Ipht3HxaepgDgSXPs2Nz0oOjvZxoCuXMQAvD_BwE

[15] Department of Health. District health plan KwaZulu-Natal 2018 [homepage on the Internet]. [cited 2020 Nov 02]. Available from: www.kznhealth.gov.za/2018-2019-annual-report.pdf

[16] Africanadvice.com. Old age homes in Durban [homepage on the Internet]. Durban: Africanadvice.com; 2021 [cited 2020 Oct 10]. Available from: https://www.africanadvice.com/Old_Age_Homes/Durban/

[17] Horowitz A, Teresi J, Cassels LA. Development of a vision screening questionnaire for older people. J Gerontol Soc Work. 1991;17(3–4):37–56. 10.1300/J083v17n03_04

[18] McGee M, Jensen GL. Mini Nutritional Assessment (MNA): Research and practice in the elderly. Nestle Nutrition Workshop Series Clinical & Performance Programme. Volume 1. Vellas B, Garvey PJ, Guigoz Y, editors. Basel: S Karger AG, Oxford University Press, 2000; 195 pages.

[19] Jupiter T, DiStasio D. An evaluation of the HHIE-S as a screening tool for the elderly homebound populatin. Journal of the Academy of Rehabilitative Audiology. 1998; 31:11–21.

[20] Graf C. The Lawton instrumental activities of daily living scale. Am J Nurs. 2008;108(4):52–62. 10.1097/01.NAJ.0000314810.46029.74 18367931

[21] Rolfson DB, Majumdar SR, Tsuyuki RT, Tahir A, Rockwood K. Validity and reliability of the Edmonton Frail Scale. Age Ageing. 2006;35(5):526–529. 10.1093/ageing/afl041 16757522 PMC5955195

[22] Parmelee PA, Katz IR. Geriatric depression scale. J Am Geriatr Soc. 1990;38(12):1379. 10.1111/j.1532-5415.1990.tb03461.x 2254577

[23] Kurlowicz L, Wallace M. The mini-mental state examination (MMSE). Thorofare, NJ: SLACK Incorporated; 1999. 10.3928/0098-9134-19990501-08 10578759

[24] Perold A, Muller M. The composition of old age homes in South Africa in relation to the residents and nursing personnel. Curationis. 2000;23(1):87–94. 11140035 10.4102/curationis.v23i1.615

[25] NHLBI. Guidelines (2013) for managing overweight and obesity in adults. Preface to the Expert Panel Report (comprehensive version which includes systematic evidence review, evidence statements, and recommendations). Obesity. 2014;22 Suppl 2:S40. 10.1002/oby.20822 24961824

[26] Ryan D, Heaner M. Guidelines (2013) for managing overweight and obesity in adults. Preface to the full report. Obesity. 2014;22 Suppl 2:S1–S3. 10.1002/oby.20819 24961822

[27] Bhaskaran K, Douglas I, Forbes H, dos-Santos-Silva I, Leon DA, Smeeth L. Body-mass index and risk of 22 specific cancers: A population-based cohort study of 5·24 million UK adults. Lancet. 2014;384(9945):755–765. 10.1016/S0140-6736(14)60892-8 25129328 PMC4151483

[28] Kasen S, Cohen P, Chen H, Must A. Obesity and psychopathology in women: A three decade prospective study. Int J Obes. 2008;32(3):558–566. 10.1038/sj.ijo.0803736 17895885

[29] Luppino FS, De Wit LM, Bouvy PF, et al. Overweight, obesity, and depression: A systematic review and meta-analysis of longitudinal studies. Archiv Gen Psychiatry. 2010;67(3):220–229. 10.1001/archgenpsychiatry.2010.2 20194822

[30] Roberts RE, Deleger S, Strawbridge WJ, Kaplan GA. Prospective association between obesity and depression: Evidence from the Alameda County Study. Int J Obes Relat Metab Disord. 2003;27(4):514–521. 10.1038/sj.ijo.0802204 12664085

